# Application of whole genome sequence analysis to the study of *Mycobacterium tuberculosis* in Nunavut, Canada

**DOI:** 10.1371/journal.pone.0185656

**Published:** 2017-10-05

**Authors:** Andrea D. Tyler, Elaine Randell, Maureen Baikie, Kym Antonation, Debra Janella, Sara Christianson, Gregory J. Tyrrell, Morag Graham, Gary Van Domselaar, Meenu K. Sharma

**Affiliations:** 1 National Microbiology Laboratory, Public Health Agency of Canada, Winnipeg, Manitoba, Canada; 2 Government of Nunavut, Nunavut, Canada; 3 The Division of Diagnostic and Applied Microbiology, University of Alberta, Edmonton, Alberta, Canada; 4 Department of Laboratory Medicine and Pathology, University of Alberta, Edmonton, Alberta, Canada; 5 The Provincial Laboratory for Public Health (Microbiology), Edmonton, Alberta, Canada; 6 Department of Medical Microbiology & Infectious Diseases, Max Rady College of Medicine, University of Manitoba, Winnipeg, Manitoba, Canada; 7 Department of Computer Science, University of Manitoba, Winnipeg, Manitoba, Canada; Michigan State University College of Veterinary Medicine, UNITED STATES

## Abstract

Canada has one of the lowest rates of tuberculosis (TB) in the world, however, among certain sub-populations, disease incidence rates approach those observed in sub-Saharan Africa, and other high incidence regions. In this study, we applied mycobacterial interspersed repetitive unit (MIRU) variable number of tandem repeat (VNTR) and whole genome sequencing (WGS) to the analysis of *Mycobacterium tuberculosis* isolates obtained from Northern communities in the territory of Nunavut. WGS was carried out using the Illumina MiSeq, with identified variants used to infer phylogenetic relationships and annotated to infer functional implications. Additionally, the sequencing data from these isolates were augmented with publically available WGS to evaluate data from the Nunavut outbreak in the broader Canadian context. In this study, isolates could be classified into four major clusters by MIRU-VNTR analysis. These could be further resolved into sub-clusters using WGS. No evidence for antimicrobial resistance, either genetic or phenotypic, was observed in this cohort. Among most subjects with multiple samples, reactivation/incomplete treatment likely contributed to recurrence. However, isolates from two subjects appeared more likely to have occurred via reinfection, based on the large number of genomic single nucleotide variants detected. Finally, although quite distinct from previously reported Canadian MTB strains, isolates obtained from Nunavut clustered most closely with a cohort of samples originating in the Nunavik region of Northern Quebec. This study demonstrates the benefit of using WGS for discriminatory analysis of MTB in Canada, especially in high incidence regions. It further emphasizes the importance of focusing epidemiological intervention efforts on interrupting transmission chains of endemic TB throughout Northern communities, rather than relying on strategies applied in regions where the majority of TB cases result from importation of foreign strains.

## Introduction

Tuberculosis (TB) is a global disease with an estimated one third of the world’s population infected with the causative agent, *Mycobacterium tuberculosis* (MTB) [1]. While global rates of this insidious infection have dropped since 2000, it remains one of the top ten sources of mortality globally [1]. In Canada, the incidence of TB is low, and has plateaued at an annual rate of 5 per 100000 persons in 2004 to 4.4 per 100000 in 2014 [2]. Despite this gradual reduction, rates of TB remain high among certain subgroups within Canada, representing a significant challenge in efforts to meet global TB elimination targets [3]. In the Canadian context, TB incidence is driven primarily by higher rates of disease among two distinct subpopulations; namely the foreign born and Indigenous Peoples [2]. These Canadian subpopulations have dissimilar epidemiological profiles, with foreign-born individuals commonly infected in their home country and displaying restricted transmission of disease within Canadian communities, versus Canadian-born Indigenous Peoples infected by endemic clonal outbreak strains circulating through communities, and contributing to maintenance of transmission chains [4–6]. Within this context, this study describes the ongoing TB epidemic in the territory of Nunavut, where, in 2003, a dramatic increase in the incidence of TB occurred, reaching a maximum rate of 299.8 per 100,000 in 2010 (Territory population of 33,353)[2]. This increase resulted in an incidence rate of 64x the Canadian national average [7]. The reason for this dramatic rise is not clearly understood and is likely multi-factorial. However, to develop targeted medical and public health interventions, understanding and describing the molecular basis of the outbreak is required, as, to date, neither the epidemic nor the circulating strains in this region have been well characterized.

The current gold standard in molecular epidemiological analysis of MTB is mycobacterial interspersed repetitive unit-variable number of tandem repeat (MIRU-VNTR). However, this method targets only small parts of the genome for investigation, and as such has lesser potential for resolving clusters than do approaches that take into account the composition of the whole genome. Further, the recent increase in cost effectiveness of next-generation sequencing for whole genome sequencing (WGS) analyses has rapidly increased the utility of this method for outbreak detection and surveillance [8,9]. To date, such studies in Canada have revealed that strictly epidemiological contact tracing analyses are hindered by the quality and completeness of data shared with health officials [10,11]. Furthermore, several of these analyses have shown that multiple, genetically distinct MTB strains may be circulating concurrently within Northern communities, breaking up what had appeared to be single outbreak clusters when investigated using traditional molecular typing strategies (ie MIRU-VNTR) [10,12]. These observations highlight the importance of high resolution molecular discrimination of strains using WGS for appropriately understanding the transmission dynamics of an epidemic, and the utility of using this information to identify and optimize public health activities aimed at interrupting and reducing transmission. Additionally, WGS data can be used to identify molecular evidence for strain-specific phenotypic variability including, but not limited to the acquisition and spread of anti-mycobacterial drug resistance.

The ongoing outbreak of TB in Nunavut, prior to this study, had been poorly characterized. To address this knowledge gap, we have applied both 24-locus MIRU-VNTR and WGS analysis to isolates collected between 2003 and 2013. The aims of this study were twofold: to characterize the amount of nucleotide-level diversity identified via WGS within the larger MIRU clusters in circulating MTB in Nunavut; and to perform a meta-analysis including our own data as well as all available Canadian MTB strains currently available in public reference databases, in order to better understand this epidemic within the Canadian context.

## Methods

### Study samples

Clinical specimens from Nunavut were sent to the Provincial Laboratory for Public Health in Edmonton, Alberta for culture and identification of MTB. Once identified, MTB isolates were sent to the National Reference Centre for Mycobacteriology (NRCM; National Microbiology Laboratory, Public Health Agency of Canada, Winnipeg, Canada) for MTB genotyping, as part of ongoing surveillance efforts being conducted in collaboration with the public health department in the territory of Nunavut. Isolates included in this study were collected as part of routine disease surveillance procedures in the Nunavut Territory. No clinical data relating to patient of origin was collected by the Public Health Agency of Canada, and permission to publish was obtained from the Government of Nunavut. A total of 274 isolates collected between 2003 and 2013, were included in genomic analyses, with a subset of 233 representative isolates undergoing WGS. Antimicrobial resistance testing was conducted by the provincial lab submitting the strains prior to their submission to the NRCM. As no first line resistance was detected, secondary testing was not performed. Several isolates were collected from individuals with multiple diagnoses of TB throughout the study time period. Five subjects had MTB isolates identified from two separate time points, and a sixth subject had three MTB isolates obtained at different time points. For all analyses, selected strains were cultured on Lowenstein-Jensen slants, using standard, aerobic growth conditions.

### MIRU-VNTR

A loopful of cultured MTB was suspended in TE buffer, heated to 100°C for 10 minutes, and sonicated (ultrasonicator)(ThermoFisher, Waltham, USA) for 15 minutes [13,14]. Lysates were then centrifuged for 2 minutes (13500xg), with the supernatant used for MIRU-VNTR analysis. Following DNA extraction, 24-locus MIRU-VNTR was performed using the 3730xl DNA analyzer (Applied Biosystems, Foster City, USA), with patterns analyzed in GeneMarker (v. 2.6.7) (SoftGenetics, LLC, State College, USA) as per the procedure described by De Beer et al [15][16]. MIRU-VNTR patterns were maintained in BioNumerics (v. 6.0) (Applied Maths, Inc., Austin, USA). Classification of an isolate into a cluster required identical 24-loci MIRU patterns. Complete 24-loci patterns could not be generated in some cases due to one or more loci having inconclusive results. Loci with missing data were imputed using the ‘mice’ package in R (v. 3.3.3)[17]. Loci which could still not be assigned were left blank, and considered unknown/missing at that position. Isolate clustering was visualized as a minimum spanning tree (MST) using PHYLOViZ [18] using the goeBURST algorithm [19]. Additionally, MIRU-VNTR based phylogenies were generated using the Gower distance, with Unweighted Pair Group Method with Arithmetic Mean (UPGMA) clustering performed using the R statistical analysis package.

### Whole genome next generation sequencing

DNA was extracted for WGS analysis using a different protocol from that used for MIRU-VNTR. Prior to DNA extraction, a loopful of bacterial culture was placed in TE buffer and heated at 100°C for 10 minutes. DNA was extracted using the MasterPure Complete DNA & RNA Purification kit by Epicentre (Illumina, Madison, USA), which includes a Proteinase K treatment to aid in digestion of the mycobacterial cell wall prior to DNA extraction. Extracted DNA was quantified fluorometrically using PicoGreen (Life Technologies, Burlington, Canada) or Qubit (Life Technologies, Burlington, Canada).

Sequencing libraries were prepared using the TruSeq Nano DNA HT Sample preparation kit (Illumina, Victoria, Canada), following manufacturer suggested protocols [20]. MTB DNA was indexed for multiplex sequencing using Illumina barcodes, and DNA was size-selected to be in the range of 600–1000 basepairs (average peak of ~800bp) using the BluePippin (Sage Science, Beverly, USA). Paired-end sequencing was performed using the Illumina MiSeq with the 600-cycle sequencing format kit (MiSeq reagent kit v.3)(Illumina, Victoria, Canada), with samples multiplexed to 24 samples per flow cell. All sequencing data is available through the SRA under bioproject PRJNA388806.

### Sequence data analysis

To determine the lineage of our samples, *in silico* spoligotyping was performed using SpolPred [21] on the raw FASTQ reads. These spoligotypes were confirmed, and subgroupings identified using the SNP typing scheme described by Coll et al [22]. Read quality filtering, reference genome-based alignment, variant calling and construction of a distance matrix were performed using the SNVPhyl pipeline implemented in Galaxy [23]. Briefly, this software performs reference mapping using SMALT (v. 0.7.5) and SAMtools [24] followed by variant calling using FreeBayes (v. 0.9.20)[25] and BCFtools [26]. Identified single nucleotide variants (SNV)s were filtered based on depth of coverage, with a minimum of 10x coverage per loci required for SNV calling. Additionally, regions with mean mapping FASTQ quality scores less than 30 were excluded. A minimum agreement of 75% of sequencing reads was required in order to confirm a variant call. SNVs falling within high density SNV regions (2 per 20 bp sliding window) or predefined areas of exclusion were not used in construction of a phylogeny. Regions excluded from analysis in this study included simple repeats identified using island viewer [27], PHAST [28] and the nucmer function in MUMmer [29], as well as MIRU-VNTR loci [16] and PE-PGRS and PPE regions (identified from NCBI annotation of the H37Rv reference genome), based on the known difficulty in accurately mapping sequences to these regions. In order to maximize the number of SNVs included in the analysis, samples in which more than 5% of identified variants could not be called due to insufficient quality were excluded (*n* = 2). Identified SNVs were then used to calculate the phylogenetic distance between isolates using the generalized time reversible (GTR) model, with PhyML [30] used to construct a phylogeny. H37Rv (NC_000962.3) was used as a reference genome, with SNV numbering as per the position of the variant along the reference genome.

Differences between MIRU clustering and SNV clustering were visually inspected using the phytools [31] package in R. Associations between SNPs and different MIRU-VNTR clusters were evaluated using Fisher’s exact test (FET) in R. Association between MIRU-VNTR and SNP clustering in the samples with both data types was also visually assessed. SNVs identified through the SNVPhyl pipeline that were associated with particular clusters, were annotated using SNPeff and annotations parsed and processed using a provided customized Perl script [32]. Annotations were manually inspected for variants occurring in AMR genes listed in the TBDream database [33]. Samples in which there was potential evidence of AMR-loci via this analysis were also run through Mykrobe predictor-TB [34] in order to confirm associations.

### Canadian MTB WGS meta-analysis

Raw FASTQ sequence data was obtained from NCBIs Sequence Read Archive (SRA), from three Canadian WGS studies of endemic Canadian TB (Accession numbers SRP046976, SRP039605, SRA020129)(S1 Methods)[10,12,35]. These sequences were analyzed in conjunction with WGS data from the Nunavut TB isolates (as described above), as part of a Canada-wide meta-analysis in order to establish a more complete picture of TB across Canada. Clusters were identified that had 90% bootstrap confidence and were a minimum genetic distance threshold of 0.015 (maximum pairwise intragroup genetic distance of 1.5%, and approximately 50 SNVs separating) and 0.0025 (approximately 10 SNVs separating), with a large cluster threshold of 10, using ClusterPicker [36] in order to identify broadly similar groups of isolates.

## Results

### Sequenced strain characteristics

*In silico* spoligotyping was used to position our sequenced strains from Nunavut in the context of other global TB data. All samples which clustered into one of the four observed groups via MIRU-VNTR (see below) had the same spoligotype pattern (777777777760771), reflecting the highly clonal nature of this outbreak. All Nunavut isolates were classified together into the Euro-Amercian lineage (Lineage 4)[37]. Additionally, using the typing scheme described by Coll et al [22], the lineage specific SNP at position 931123 (against H37Rv NC_000962.3), confirmed that all Nunavut isolates indeed belonged to Lineage 4 with 218 positive for both 4.1 and 4.4-specific markers (62657, 4307886), 14 positive for 4.4 and 4.8 markers (4307886, 3836739), 1 positive for markers 4.1 and 4.8 (62657, 3836739).

### MIRU-VNTR

**Fig 1:**
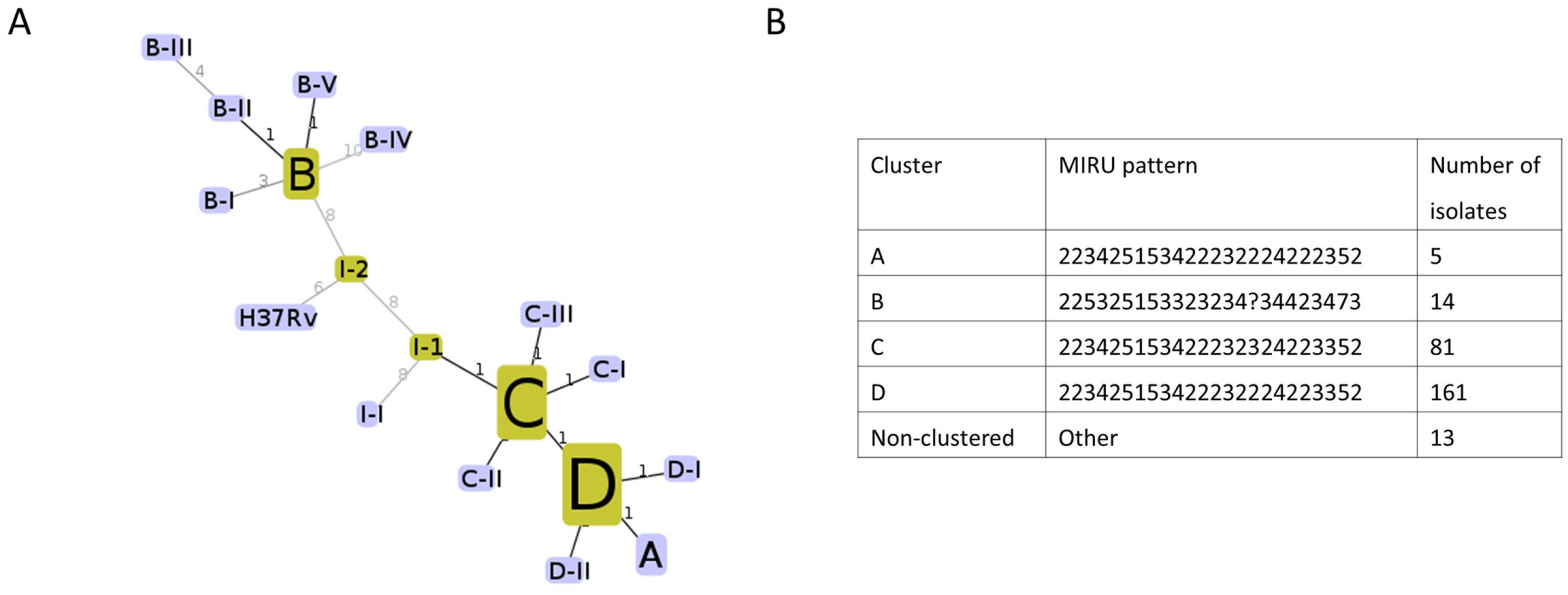
Description of the MIRU clusters identified in the cohort of samples included from Nunavut. A) Minimum Spanning Tree (MST) showing the relationship between identified MIRU patterns in this cohort B) Number of isolates in each of the four main MIRU clusters. MIRU loci patterns are in the order: 154, 580, 960, 1644, 2059, 2531, 2687, 2996, 3007, 3192, 4348, 802, 424, 577, 1955, 2163, 2165, 2347, 2401, 2461, 3171, 3690, 4052, 4156, with numbering as per the MIRU-VNTRplus database [16,38].

24-loci MIRU-VNTR performed on the 274 isolates from this region identified four clusters based on 100% pattern identity (Fig 1). The two largest clusters (C & D) were separated by a single difference at locus 2163. The third cluster (B) differed from C and D by several additional loci and was observed more commonly in a population which was geographically isolated from the larger two clusters. Cluster A was the smallest, detected in only five individuals. This cluster differed from clusters C and D at a single loci, and was confined to a single geographic area. Eight additional isolates with unique MIRU patterns remained unclustered.

### SNV typing of Nunavut strains based on WGS sequencing data

Of the 233 isolates that underwent WGS, 231 had sufficient data available for inclusion in the analysis, while two did not have sufficient coverage for confident SNV calling. In total, 2109 SNVs were identified when compared to the H37Rv reference genome of these, 1697 met our criteria for inclusion in the core genome representation and were subsequently included in phylogeny construction. The major clusters observed by MIRU were also observed in the WGS data (S1 Fig). However, WGS data provided enhanced resolution, breaking up large MIRU clusters based on the greater number of phylogenetically informative sites contained within the SNV dataset (S1 Fig). The improved clustering resolution obtained via WGS corresponded well in most cases with the geographical region of specimen origin.

### SNV cluster analysis and variant annotation

Intra- and inter-cluster SNV differences characterizing the four main MIRU clusters are depicted in Table 1. Over 1200 SNVs were detected between the four main MIRU clusters, with the maximum SNV difference between any pair of clusters totaling 784. The intra-cluster variability was also evaluated, with MIRU cluster B showing the largest amount of SNV variability compared to the other dominant clusters (Table 1; S1 Fig). Variant functional annotation predicts that several of the SNVs identified may have a large impact on cellular processes specifically in relation to pathways associated with pathogen-host interactions. Detected nonsense mutations that were present in a large fraction of the population are included in Table 2, while all detected SNVs and their annotations are described in S1 Table. Of the nonsynonymous variants that had predicted functional outcomes, several were common across all of our isolates (in comparison to the H37Rv reference), suggesting common alterations to bacterial physiology and function. Our data identified additional variants that characterized particular sub-clusters in this dataset. None of the variants identified in this analysis, to our knowledge, have been previously associated with MTB antimicrobial resistance (AMR). One SNV identified in our cohort among seven MTB strains was a Val981Leu variant in *embC*; a gene that has previously been associated with ethambutol resistance, but only when found in conjunction with mutations in *embA* [39]. As expected no phenotypic resistance to this antibiotic was reported among these or any of the other analyzed strains. No additional mutations previously associated with AMR as reported in either the TBdream database [40], or Mykrobe Predictor [34] were detected in our cohort.

**Table 1:** Pairwise intra- and inter-cluster SNV variability in four MIRU groups.

**MIRU Group**	**A (n = 5)**	**B (n = 14)**	**C (n = 64)**	**D (n = 140)**
**A**	0.6 (0–2)			
**B**	766 (749–773)	12.4 (0–65)		
**C**	52.9 (36–57)	784.1 (751–794)	3.5 (0–42)	
**D**	8.7 (1–53)	763.9 (745–790)	49.9 (0–59)	9.1 (0–55)

**Table 2:** Nonsense single nucleotide variants (resulting in premature stop, or abrogation of start). Loci based on genomic position and numbering in the H37Rv reference genome (NC_00962.3). All described alternate alleles at specified loci are in relation to the reference sequence at that position. Included loci are those with at least 5 isolates possessing the variant genotype. All were significantly associated with a MIRU cluster (p_FDR_ <0.05).

SNV locus on H37Rv reference	Gene/locus name	Alternate allele	Variant allele MIRU A	Variant allele MIRU B	Variant allele MIRU C	Variant allele MIRU D
142246	*oxcA*	T	0	0	61	2
212244	Rv0180c	A	5	0	62	140
234477	Rv0197	G	5	14	62	140
707337	Rv0613c	A	0	14	0	0
1037911	*pstA1*	T	5	14	62	140
1989043	*cut1*	C	4	0	39	107
1989044	*cut1*	A	4	0	39	107
2125341	*bfrA*	C	0	14	0	0
3356519	Rv2997	A	0	12	0	0
3689523	*lpdA*	T	5	14	62	140
3870808	*mycP4*	T	5	0	62	140
3959957	*ltp3*	A	0	14	0	0
3966813	Rv3529c	A	0	14	0	0
4365461	*eccA2*	A	0	14	0	0

### Comparison isolates from repeat sampling from individuals at multiple times

**Fig 2:**
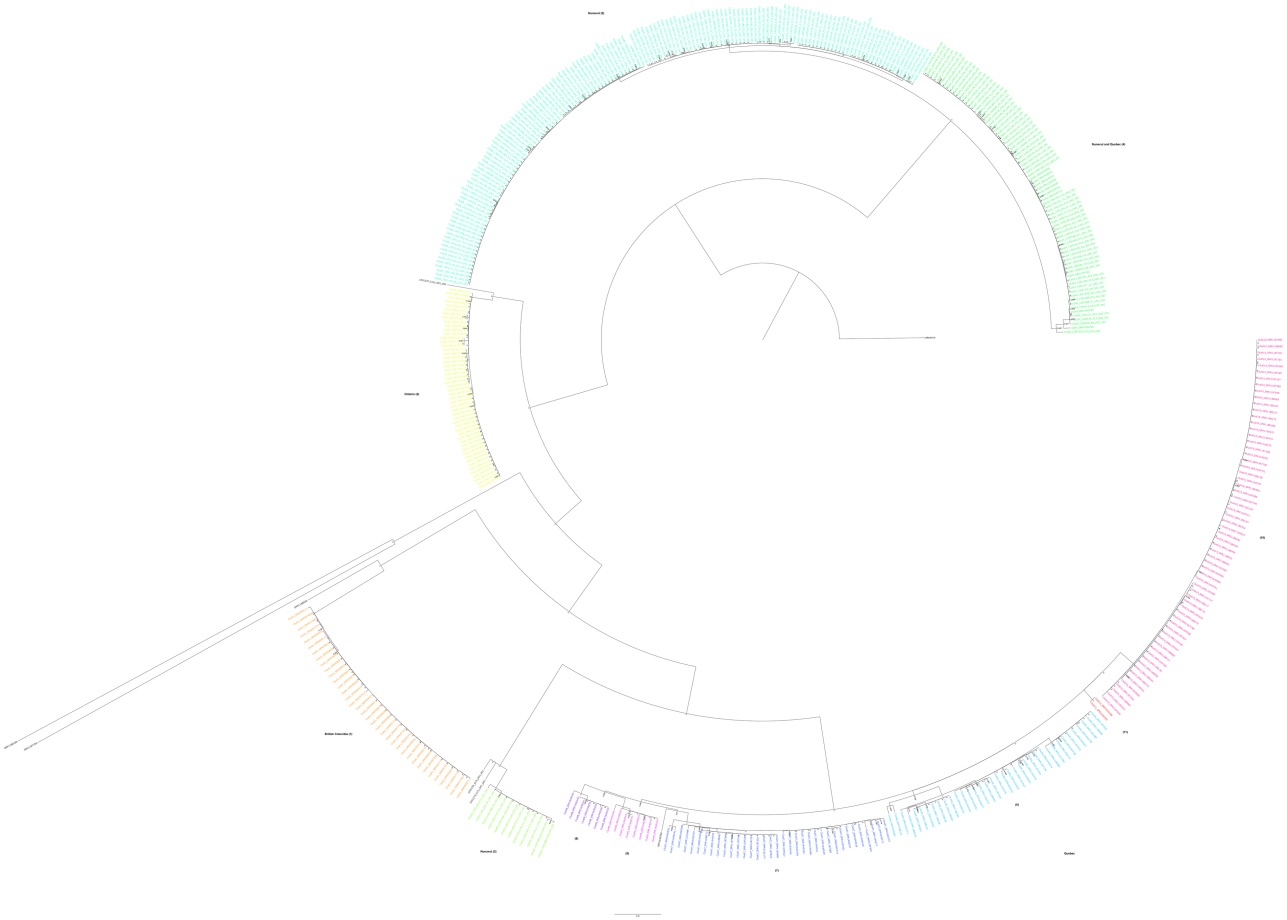
Phylogeny depicting isolate relatedness based on WGS data, including both isolates from Nunavut, as well as those previously reported from other Canadian regions. Colouring of the major clusters was performed using ClusterPicker with a maximum genetic distance threshold of 0.015, with predominant geographic location and WGS cluster number included beside the corresponding cluster. Isolates not coloured represent individual samples that could not be grouped with any of the major clusters at this distance threshold.

Isolates obtained from individuals at multiple time points (a total of 13 strains in 6 subjects) (Table 3) suggested possible incomplete treatment of disease in 4 of 6 cases, although reinfection with another strain from the same cluster cannot be ruled out. Molecular evidence suggested that re-infection with a completely distinct strain had occurred in one subject (subject 1). In this individual, MIRU-VNTR samples differed from each other at two loci. In our SNV-based analysis, samples from this individual differed by 53 SNVs. Two separate isolates from subject 2, meanwhile, had identical 24-locus MIRU-VNTR patterns; however differed from each other by 10 SNVs via genomic analysis. Furthermore, each of the isolates from subject 2 were more closely related to an isolate from another subject in a separate sub-cluster within the larger WGS dataset (Fig 2).

**Table 3:** Repeat sampling of individuals from whom multiple isolates were obtained.

Individual	Samples	Years separating samples	Sample source	MIRU cluster	Number of SNVs separating
1	1001198^a^-1001255^b^	1	unknown	A^a^;C^b^	53
2	1001226^a^-1300352^b^	3	sputum	D	10
3	1300361–1300384	3	sputum	D	3
4	1001230–1300312	5	sputum	D	1
5	1001263^a^-1100021^b^-1300300^c^	5months^a,b^; 5^a,c^	sputum	D	0^a,b^; 1^b,c^; 1^a,c^
6	1100296–1300383	0	sputum	C	0

For cases in which isolates have different clustering patterns, superscript letters denote which isolates are referred to in the subsequent columns.

### Meta-analysis of publically available Canadian MTB

In addition to the isolates sequenced as part of this study, WGS data on 292 MTB strains identified from other Canadian regions (Ontario (n = 87), Quebec (n = 169) and BC (n = 36)) were obtained from the SRA in NCBI, with a sum of 257 that met coverage and quality requirements for inclusion in the meta-analysis. In total, 5614 SNVs were identified in this collection among all isolates when using the H37Rv genome as reference. Following filtering, 3830 high quality core SNVs were used to construct the phylogeny. Cluster generation using ClusterPicker at a genetic distance threshold of 0.015 (corresponding to approximately 50 SNVs separating) and 0.0025 (corresponding to approximately 10 SNVs separating), identified 11 and 28 distinct clusters respectively, as well as a small number of individual strains that did not cluster with any of the larger groups. Among the more distant clustering (0.015), Nunavut strains were found in three large clusters, with several isolates failing to cluster. WGS SNV data demonstrated that Nunavut strains clustered separately from those which were previously described in other Canadian communities with one notable exception (Fig 2): a set of isolates which originated in the Nunavik region of Northern Quebec were found to cluster with a subset of the Nunavut isolates (n = 69; WGS Cluster 4). The communities of origin for these specimens were geographically situated directly across a large body of water from each other. Inter and intra-cluster SNV variation is described in S2 Table. The maximal mean intra-cluster difference was 13.7 (range 0–46) SNVs, and inter-cluster 785.9 (range 767–794). Unsurprisingly, the largest differences were observed between several Canadian samples and the H37Rv reference, with mean SNV differences of 597.6 (range 455–1191).

When examining the SNV data using a cluster threshold of 0.0025 (10 SNV maximum difference), 28 clusters were generated with 98 strains not falling into any of these clusters. Nunavut isolates continued to cluster more closely with other isolates from this region than those from the rest of Canada. Interestingly, the isolates grouped together in cluster 4 (Nunavut and Northern Quebec), remained clustered even at this increased resolution threshold. Among the Nunavut isolates, seven smaller clusters were generated in addition to the three larger clusters described above (S2 Fig).

## Discussion

This study employed both 24 locus MIRU-VNTR and WGS to investigate a TB outbreak occurring in the Northern Canadian territory of Nunavut, between 2003 and 2013. Among the isolates included for analysis, MIRU-VNTR results suggested that TB in this region is highly clonal with four patterns dominating the outbreak. WGS data supported these results yet provided additional resolution, demonstrating that a majority of circulating strains are highly genetically related, with the exception of those in MIRU cluster B. There were, however, several isolates showing greater genetic distance via WGS than by MIRU, with SNV differences extending to a level that would be unlikely within a population of epidemiologically related strains [41].

Based on our genomic findings we can speculate that the TB outbreak in Nunavut is unlikely to have arisen as a result of a recent introduction of foreign strain types. We hypothesize, therefore, that new TB introduction to this population has occurred relatively infrequently and that increased detection of TB throughout this region between 2003 and 2013 is predominately driven by increased spread of circulating endemic MTB strains. Supporting this, a previous study by Pepperell et al showed, historically, that there was greater diversity among MTB strains in Quebec, with increased homogeneity in more remote regions (ie. Saskatchewan) along routes of commerce (ie the fur trade) [42]. Furthermore, Nunavik MTB strains (Northern Quebec), appear to be quite similar to several of those from Nunavut [9]. Therefore, while Nunavut samples were not included in the Pepperell analysis, our analysis shows they are congruent with these findings, namely that their low genomic variability is consistent with few introductory events in the human population.

Previous WGS studies of MTB have demonstrated that globally, the maximum genetic distance between any two human strains of MTB is approximately 1800 SNVs [43–45]. Our results show 1191 SNVs separating Canadian strains from the H37Rv reference genome, and maximum inter cluster (SNV phylogeny) difference of 785, are in keeping with previous descriptions of lineage-specific mutation rates [45], and in our case may be indicative of a relatively recent introductory event or reemergence of disease (during a period of lowered TB awareness) in communities in Northern Canada, similar to that reported by Bjorn-Mortensen et al in Greenland [46].

Of note is previous work suggesting that in culture, a lower rate of mutation of lineage 4 strains results in a lower rate of acquisition of drug resistance-associated variants [47]. The lack of detection of resistance-associated polymorphisms in our MTB cohort, seems to agree with this observation. Further investigation of strains in this region, and long term surveillance for the development of antimicrobial resistance may be warranted in order to address this question.

The comparatively low number of SNVs separating the majority of Nunavut isolates, while interesting from a molecular biology perspective, complicates analyses. Our data examining a small subset of isolates obtained longitudinally from individuals highlights this challenge, as determining whether disease occurred as a result of reactivation of untreated disease or instead via reinfection was not easily elucidated in all cases. Numerous studies have attempted to establish numerical cutoffs in detected SNVs that could be used for differentiating reinfection from relapse [48]. Such cutoffs may have utility in situations wherein circulating strains are not highly polymorphic and the probability of reinfection by a closely related strain is very low. However, in the context of Northern Canadian TB, application of a similar numerical cutoff would require validation, with thresholds likely differing from those generated for analyses conducted in other locales and using alternative computational methods. This would suggest that application of a single, universal threshold to inform cluster analysis and subsequent contact tracing is not feasible, especially given that the likelihood of reinfection with a closely related strain is dependent upon the structure of the outbreak, and the host and bacterial population in question. In the context of our own data, in a subject with multiple episodes of active TB disease separated by three years, ten SNVs differentiated the collected isolates. Although ten SNVs is within the range previously reported to occur in a similar timeframe (0.5 SNVs per genome per year)[49,50], it exceeds the maximum rate predicted by other studies [9], and is unlikely among samples collected in the shorter three year timescale under study. Additionally, the observation that each of the isolates clustered independently with a separate set of strains, leads us to speculate that the subject in question (individual 2) was infected with distinct MTB on two separate occasions. In this case, the added information provided by WGS in comparison to MIRU is instructive, and in conjunction with additional epidemiological data, may be useful for investigations of common sources of infection, rates of disease development (from infection to diagnosis), and issues related to acquisition of immunity to MTB infection in this host population.

One factor not considered in this analysis is the potential for mixed infections, or differential microevolution of subpopulations of MTB within individual patients. Several previous studies have demonstrated that multiple genetically distinct MTB strains resulting from novel infection events may be found within a single individual [51,52]. Although we do not have any evidence of mixed infections due to our chosen methodology, it is known that host selective pressures in conjunction with antibiotic treatment, may contribute to acquisition of novel genetic variants, including mutations associated with drug resistance [43,53,54]. Future studies in which WGS is performed directly on patient material and/or analysis of rare variants among closely related strains from the same subject is applied, may provide greater insight regarding the role of mixed infections in this Canadian populations.

The high quality of WGS data obtained in this cohort allowed us to conduct a more detailed analysis investigating potential physiological implications of polymorphic loci. We did not detect any mutations known to be associated with antimicrobial resistance in MTB, nor was there evidence of the presence of other variants that may decrease drug susceptibility among this group of isolates. Notably, several variants inferred to either alter protein structure, or expression were detected. Among these were several genes previously identified as essential for MTB growth in macrophages, and virulence [55–57]. A loss in start of the *bfrA* gene among isolates in (MIRU-VNTR) cluster B was detected. This gene encodes bacterioferritin, one of two iron storage proteins in MTB. This molecule aids in maintaining iron homeostasis, although unlike ferritin (*bfrB*) is not required for survival [58]. While not directly observed to influence survival and persistence during chronic infection [58], loss of *bfrA* may have a modifying effect on bacterial fitness within the host specifically during active TB, resulting in less robust transmission of strains containing this polymorphism when compared to strains lacking the variant. The low number of isolates with this variant (total 14 isolates (5%); exclusive to MIRU-VNTR cluster B) supports this hypothesis. Also detected only in MIRU cluster B was a premature stop mutation in Rv0180c at the third amino acid of the coding sequence—effectively eliminating transcription of this molecule. This gene encodes a previously described probable transmembrane protein that is involved in interaction with and invasion of monocytes and alveolar epithelium in the members of the MTB complex [59]. Our finding of SNVs associated with abrogated production of Rv0180c, demonstrate that in these clinical strains of MTB, infection has occurred in the absence of this molecule. This suggests that there is functional redundancy within the MTB genome that allows strains to replicate in macrophages without a functional copy of this gene, although potentially at a cost to fitness. Future work investigating transmission rates of such variants may be warranted, however, our findings of several non-synonymous SNV variants is in keeping with previous work [60,61]. This may suggest that these variants have, at most, minor impacts on fitness, and do not significantly contribute to alterations in pathogenesis, virulence or host-microbial interactions.

The utility of WGS for outbreak surveillance has been clearly demonstrated by our own work as well as that of others [10,42]. The addition of epidemiological data and social network analysis to our own WGS, would improve contact tracing analyses and evaluation of the transmission dynamics of this epidemic. However, large studies of disease that span several jurisdictions are often limited in the amount of clinical and epidemiological data available. For these reasons, this study is the first to characterize TB in Nunavut, and to place this data within the known Canadian context. By comparing this data set together with studies previously carried out in populations in BC, Ontario (harbouring a diverse homeless population) and Nunavik Quebec (mostly Inuit), herein we add to the body of literature describing this topic. As it is becoming possible to concurrently evaluate greater amounts of WGS data, large studies fully describing the complete picture of TB across all of Canada, will be of use in longitudinally tracking TB epidemics, as well as in assessing risks of transmission both within and between communities. In the future, a more detailed investigation of sub-clusters identified via WGS in the context of epidemiological data, will also be valuable.

## Supporting information

S1 Methods Description of the Canadian WGS sequence data included as part of this study. (DOCX)

S1 Fig Comparison of MIRU-VNTR clustering (left) and WGS clustering (right). (PNG)

S2 Fig Phylogeny depicting isolate relatedness based on WGS data, including both isolates from Nunavut, as well as those previously reported from other Canadian regions. Colouring of the major clusters was performed using ClusterPicker with a maximum genetic distance threshold of a) 0.25% (~10 SNVs) and b) 0.5% (~20 SNVs). Isolates not coloured represent individual samples that remained distinguishable from all major clusters at this genetic distance threshold.
(TIF)

S1 Table Functional annotation of SNVs identified in Nunavut isolates inferred using SNPEff. (XLSX)

S2 Table Intra and inter-cluster SNV differences between WGS clusters identified using ClusterPicker. (XLSX)
