# Supplementary material for: Application of whole genome sequence analysis to the study of Mycobacterium tuberculosis in Nunavut, Canada
Source: PLoS One. 2017 Oct 5;12(10):e0185656. doi: 10.1371/journal.pone.0185656 (PMC5628838; doi:10.1371/journal.pone.0185656)
Supplement: S1 Methods — (DOCX) [file pone.0185656.s001.docx]

Supplementary data:

Sample selection:

Mehaffy[12] – Reports that WGS was successfully performed on 56 isolates from 53 TB cases. WGS from 55 samples available under project PRJNA259657. Of these, 51 met quality inclusion criteria for this project and were included in the phylogeny construction. Basic information on isolates, including whether the isolate could be classified as a natural mutant (based on the presence of large deletion) or wild-type (no deletion), year of collection and original sample source were available. Samples were sequenced using the Illumina MiSeq with 150-bp paired-end reads.

Lee[34] – Reports 163 genomes sequenced from Nunavik region in Northern Quebec. 169 samples were available from PRJNA240330 in the NCBI. 169 samples were downloaded, successfully passed quality control and were included in the analysis. Collection year and lineage were provided and made publically available. Samples were sequenced using the Illumina MiSeq, with 250-bp paired-end reads.

Gardy[10] – Reports 36 isolates whole genome sequenced, all of which were available through the SRA (PRJNA49659) and met our inclusion threshold. No additional information was made publically available. Sequencing was conducted using the Illumina Genome Analyzer II, with 50-bp paired end reads.
