# Supplementary figures and images for: Application of whole genome sequence analysis to the study of Mycobacterium tuberculosis in Nunavut, Canada
Source: PLoS One. 2017 Oct 5;12(10):e0185656. doi: 10.1371/journal.pone.0185656 (PMC5628838; doi:10.1371/journal.pone.0185656)

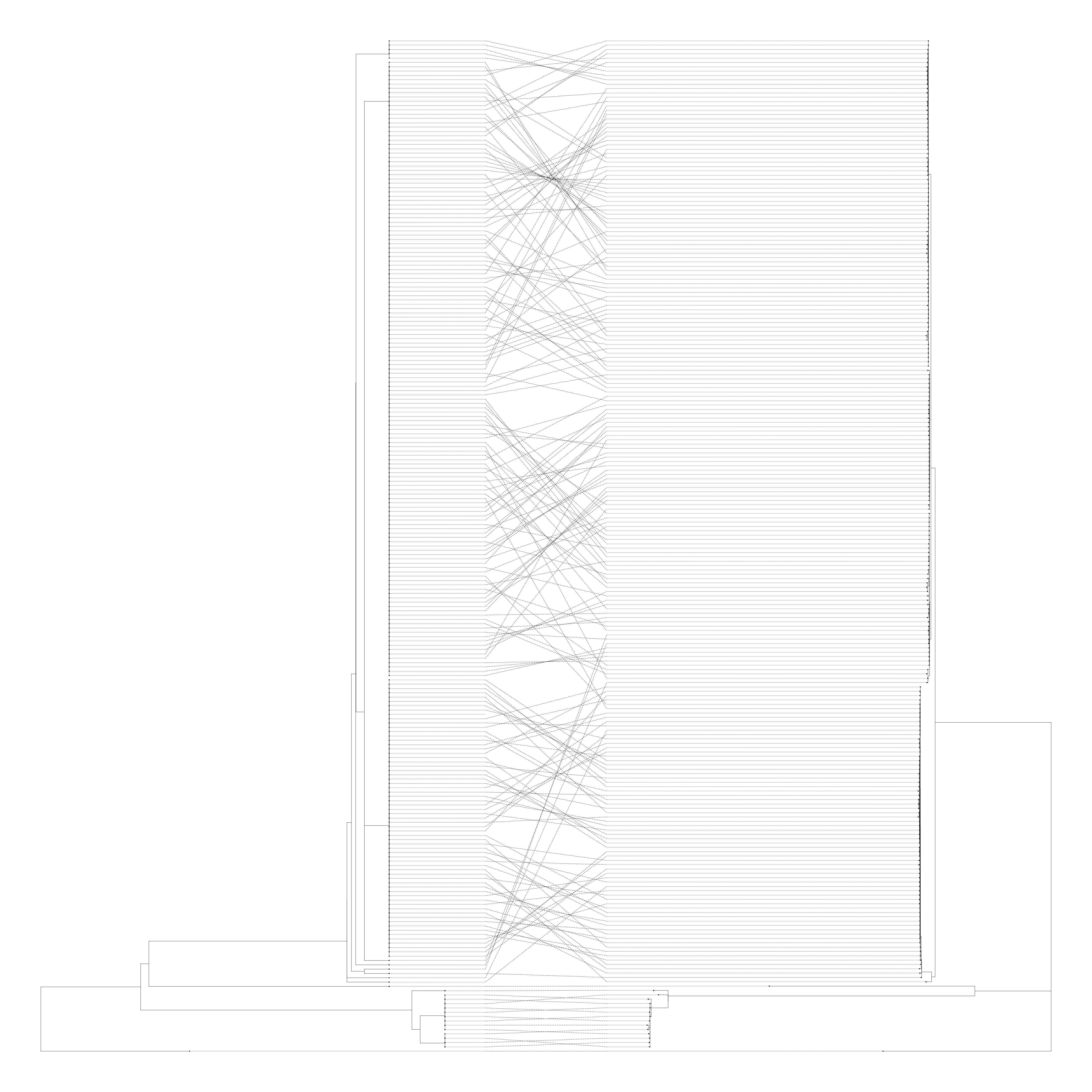

Supplement: S1 Fig — (PNG) [file pone.0185656.s002.png]

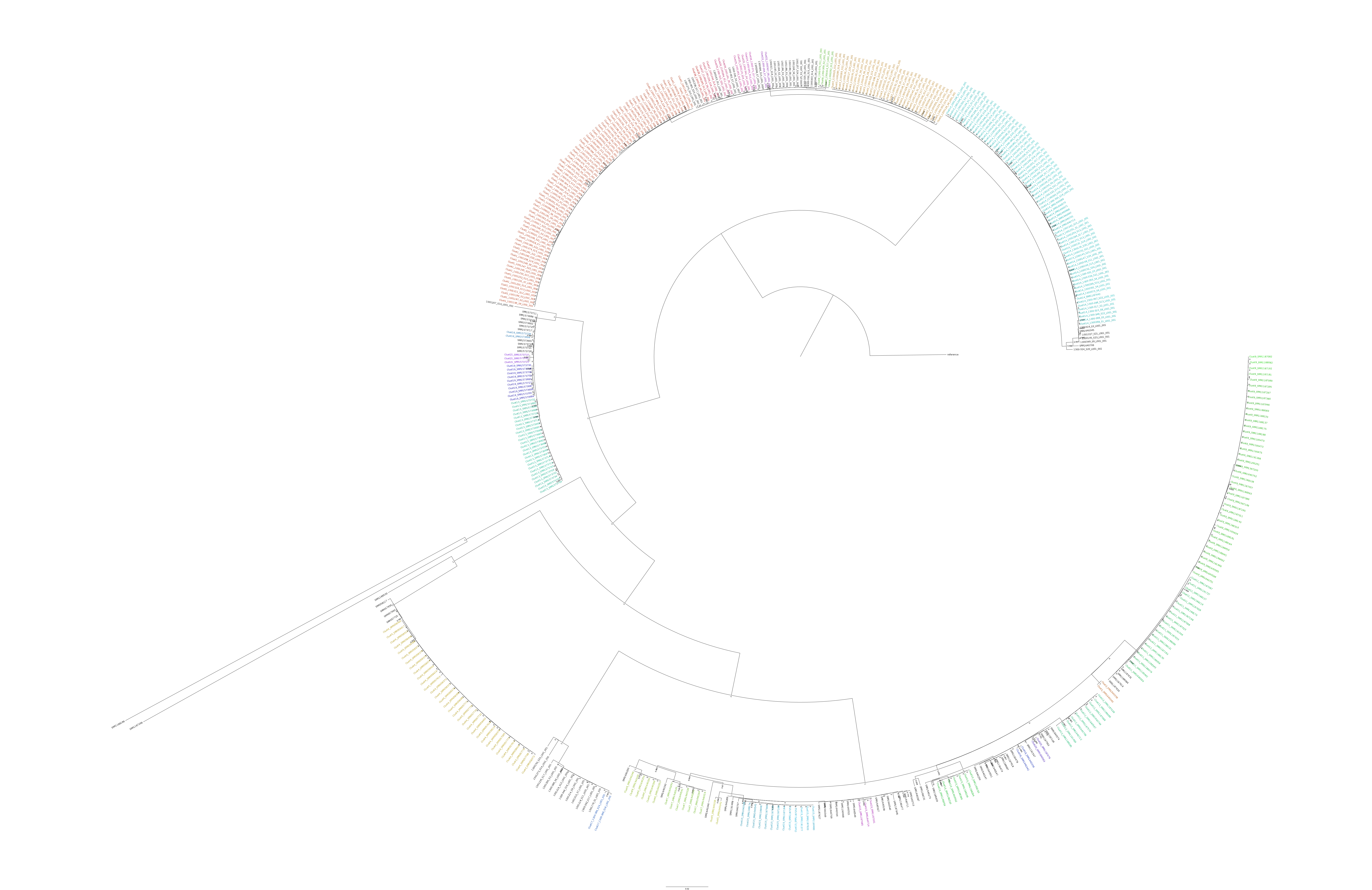

Supplement: S2 Fig — Colouring of the major clusters was performed using ClusterPicker with a maximum genetic distance threshold of a) 0.25% (~10 SNVs) and b) 0.5% (~20 SNVs). Isolates not coloured represent individual samples that remained distinguishable from all major clusters at this genetic distance threshold. (TIF) [file pone.0185656.s003.tif]
